# Lymph Node Parameters Predict Adjuvant Chemoradiotherapy Efficacy and Disease-Free Survival in Pathologic N2 Non-Small Cell Lung Cancer

**DOI:** 10.3389/fonc.2021.736892

**Published:** 2021-09-17

**Authors:** Chen-Chen Zhang, Run-Ping Hou, Wen Feng, Xiao–Long Fu

**Affiliations:** ^1^Department of Radiation Oncology, Shanghai Chest Hospital, Shanghai Jiao Tong University, Shanghai, China; ^2^School of Biomedical Engineering, Shanghai Jiao Tong University, Shanghai, China; ^3^Department of Radiation Oncology, Fudan University Shanghai Cancer Center, Department of Oncology, Shanghai Medical College, Fudan University, Shanghai, China

**Keywords:** non-small cell lung cancer, pathologic N2, disease-free survival, adjuvant chemoradiotherapy, nomogram

## Abstract

Pathologic N2 non-small cell lung cancer (NSCLC) is prominently intrinsically heterogeneous. We aimed to identify homogeneous prognostic subgroups and evaluate the role of different adjuvant treatments. We retrospectively collected patients with resected pathologic T1-3N2M0 NSCLC from the Shanghai Chest Hospital as the primary cohort and randomly allocated them (3:1) to the training set and the validation set 1. We had patients from the Fudan University Shanghai Cancer Center as an external validation cohort (validation set 2) with the same inclusion and exclusion criteria. Variables significantly related to disease-free survival (DFS) were used to build an adaptive Elastic-Net Cox regression model. Nomogram was used to visualize the model. The discriminative and calibration abilities of the model were assessed by time-dependent area under the receiver operating characteristic curves (AUCs) and calibration curves. The primary cohort consisted of 1,312 patients. Tumor size, histology, grade, skip N2, involved N2 stations, lymph node ratio (LNR), and adjuvant treatment pattern were identified as significant variables associated with DFS and integrated into the adaptive Elastic-Net Cox regression model. A nomogram was developed to predict DFS. The model showed good discrimination (the median AUC in the validation set 1: 0.66, range 0.62 to 0.71; validation set 2: 0.66, range 0.61 to 0.73). We developed and validated a nomogram that contains multiple variables describing lymph node status (skip N2, involved N2 stations, and LNR) to predict the DFS of patients with resected pathologic N2 NSCLC. Through this model, we could identify a subtype of NSCLC with a more malignant clinical biological behavior and found that this subtype remained at high risk of disease recurrence after adjuvant chemoradiotherapy.

## Introduction

Lung cancer remains the leading cause of cancer death globally (1). Non-small cell lung cancer (NSCLC) accounts for more than 80% of all lung cancer patients (2). Approximately one-fifth of NSCLC patients are classified as stage III disease (3). For resectable stage III NSCLC, surgical resection remains the main option of curative therapy, yet 5-year overall survival (OS) ranges from 16% to 42% (4, 5). Local failure and distant metastasis can occur after surgery for patients with completely resected pathologic N2 (pN2) NSCLC. The risk of locoregional recurrence is as high as 20%–40%, and the distant metastasis rate is more than 65%, which reveals the prognostic heterogeneity of this population (6–8).

The treatment management of pN2 NSCLC is still highly controversial (9, 10). Prior studies have shown that postoperative chemotherapy (POCT) is significantly associated with survival benefits (7, 11). However, the role of postoperative radiotherapy (PORT) remains an issue under debate. Some previous studies indicate benefits from PORT, while others suggest detrimental effects (12–17).

The heterogeneity observed in survival outcome suggests the inadequacy of existing treatment for the part of the patients. Emerging molecularly targeted therapy and immunotherapy have further expanded treatment options for pN2 NSCLC (18–21). However, it remains a challenge to identify patients who may benefit from specific treatments. Further research is needed to explore whether variables associated with prognosis can give recommendations for the treatment measures.

Clinical and pathologic variables such as age, tumor size, and histology have been elucidated to be related to the survival of patients with pN2 NSCLC (22–26). Several lymph node parameters have been proved to be critical for the prognosis (25, 27–31). Prior literature data show that greater lymph node ratio (LNR) was associated with a worse prognosis (29, 30). Several studies have shown that skip N2, which is defined as the tumor “skips” over the N1 (bronchopulmonary or hilar lymph node metastasis) stage to N2 (ipsilateral mediastinal lymph node metastasis) stage) had superior survival (25, 31). Recent evidence indicates that involved N2 station numbers are also a factor that has an impact on survival (31). Those parameters reflect N categories from different perspectives. However, few studies incorporated adequate lymph node information. The possible reason for this might be the limitation of open databases and the lack of a proper way to handle the multiple-collinearity among factors for building the multivariate Cox regression model.

Adaptive Elastic-Net is an ideal oracle-like method that can better handle the collinearity problem. It can incorporate the sparse processing of high-dimensional variables and select important variables from numerous variables (32). Nomograms have been recognized as a reliable and robust tool for quantifying individualized risk and predicting survival outcomes by combining and illustrating significant prognosis variables (33, 34).

This study aimed to develop an adaptive Elastic-Net nomogram with integrated lymph node parameters for resected pN2 NSCLC to predict prognosis and guide the layout of treatments individually.

## Methods

### Patient Population and Data Processing

Patients with pathologic T1-3N2M0 NSCLC in the Shanghai Chest Hospital from 2012 to 2016 were identified as the primary cohort. Patients who underwent complete resection with microscopically tumor-free resection margins were included in this study. The standard surgical method of lymph node dissection is defined as systematic nodal dissection (a dissection of three mediastinal nodal stations) or complete lymph node dissection (35). Pathologic staging was characterized according to the TNM classification in the Union for International Cancer Control (UICC) 8th ed. Patients with adjuvant therapy were treated to platinum-based POCT and or PORT (50 Gy/25 Fx or 50.4 Gy/28 Fx). Three-dimensional conformal radiotherapy or intensity-modulated radiotherapy was commonly used for performing PORT. The inclusion and exclusion criteria are described in detail in the CONSORT diagram (**Supplementary Figure 1**).

Three-quarters of patients in the primary cohort were randomly assigned to the training set. The remaining one-quarter of patients were utilized as the validation set 1. The external validation set 2 was collected from the Fudan University Shanghai Cancer Center to test the performance of the model. We identified patients from 2005 to 2012 diagnosed as pathologic T1-3N2M0 NSCLC with the same inclusion and exclusion criteria. Patients were regularly followed up every 3 months after surgery during the first 2 years. Clinical examination, enhanced chest computed tomography scans, brain magnetic resonance imaging, and ultrasonography of the abdomen were generally evaluated. Follow-up information for all patients was obtained from their most recent electronic medical review and telephone surveys. Demographic data, pathologic data, and treatment-related data were extracted. Primary tumor size was categorized as less than 3 cm, more than 3 cm and less than 5 cm, and more than 5 cm. Histology was dichotomized as squamous carcinoma and non-squamous non-small-cell lung cancer. The pathologic grade was categorized as well-differentiated, moderately differentiated, poorly differentiated, and undifferentiated. Skip N2 was defined as the tumor “skips” over the N1 (bronchopulmonary or hilar lymph nodes metastasis) stage to N2 (ipsilateral mediastinal lymph nodes metastasis) stage. LNR was defined as the number of positive nodes/the number of resected nodes and transformed into categorical variables based on quartering.

The primary endpoint was disease-free survival (DFS). DFS was defined as the time from the surgery date to the date of first locoregional recurrence, distant metastasis, or died from any cancer causes. If patients were alive at the last contact, lost during follow-up, or died from any non-cancer causes, they were censored at the last confirmed contact date. This study was approved by the institutional review board in the Shanghai Chest Hospital and the Fudan University Shanghai Cancer Center.

### Statistical Analysis

Univariate analysis was performed to estimate the effect of each clinicopathologic factor using the Kaplan–Meier methods, and p-values were derived by the log-rank test (36). Variables with a p-value less than 0.1 were incorporated into the multivariable analyses *via* the adaptive Elastic-Net Cox regression model (37). The Elastic-Net Cox regression model refers to a penalized Cox’s proportional hazards model with adaptive Elastic-Net regularization. The model uses the included clinical factors (x) as input variables and the corresponding survival outcomes (time, event) as response variables (y). The regression model would finally output the hazard of each patient (37). Hyperparameter tuning was based on cross-validation in the training set. The proportionality assumption was examined to be satisfied with log–log plots and the Cox–Snell residuals (38). The nomogram was built based on the adaptive Elastic-Net Cox regression model. The model performance was evaluated with 1,000 bootstrap resamples in the internal validation, and the external validation was performed with two validation sets (39–41). The time-dependent receiver operating characteristic (ROC) curves of the nomogram were plotted (42). Discriminability was evaluated by time-dependent area under the ROC curve (AUC) every half year from the first year to the fifth year (43). Calibration curves of the nomogram for 1-year DFS, 3-year DFS, and 5-year DFS compared the predicted survival with the observed survival. The Kaplan–Meier methods and log-rank tests were used to build survival curves for different risk groups. We determined the cutoff value as the tertile of risk points. Statistical analysis was performed by using SAS 9.4 (SAS Institute, Cary, NC) and R version 3.6.1 software (http://www.r-project.org).

## Results

### Patient Clinicopathologic Characteristics

The primary cohort consisted of the entire 1,312 patients who met the eligibility criteria. We utilized 985 patients from the primary cohort as a training set. The remaining 327 patients comprised the validation set 1. A total of 357 patients were identified according to the screening criteria from center II as an external validation cohort (validation set 2).

During a median follow-up time of 50.7 months (95% CI, 49.6 to 53.2), there were 668 events (disease recurrence) in the training set. The median follow-up time was 60.8 months (95% CI, 56.7 to 66.4) in the validation cohort, and 284 patients experienced disease recurrence during the follow-up period. Baseline characteristics of the training set, validation set 1, and validation set 2 with median survival time are listed in **Table 1**.

**Table 1:** Baseline demographics and clinical characteristics of training set and validation sets.

Variable	Training set (N = 985)	Validation set 1 (N = 327)	Validation set 2 (N = 357)
	No. of patients (%)	No. of patients (%)	No. of patients (%)
Age at diagnosis, years			
Median (IQR)	60 (55, 66)	61 (53.5, 66)	59 (53–65)
Sex			
Female	403 (40.9)	153 (46.8)	138 (38.7)
Male	582 (59.1)	174 (53.2)	219 (61.3)
Smoking history			
Never smoker or Light ex-smoker	523 (53.1)	190 (58.1)	180 (50.4)
Ex-smoker (non-light)	462 (46.9)	137 (41.9)	177 (49.6)
Family cancer history			
Without	784 (79.6)	232 (70.9)	NA
With	201 (20.4)	95 (29.1)	NA
Resection type			
Lobectomy	878 (89.1)	287 (87.8)	311 (87.1)
Sleeve resection	51 (5.2)	18 (5.5)	12 (3.4)
Pneumonectomy	56 (5.7)	22 (6.7)	34 (9.5)
Tumor size, cm			
≤3	305 (31)	96 (29.4)	148 (41.5)
>3 and ≤5	529 (53.7)	177 (54.1)	135 (37.8)
>5	151 (15.3)	54 (16.5)	74 (20.7)
Tumor location			
Right upper lobe	300 (30.5)	101 (30.9)	93 (26.1)
Right middle lobe	66 (6.7)	22 (6.7)	23 (6.4)
Right lower lobe	203 (20.6)	72 (22)	55 (15.4)
Left upper lobe	240 (24.4)	82 (25.1)	95 (26.6)
Left lower lobe	146 (14.8)	40 (12.2)	56 (15.7)
Others	30 (3)	10 (3.1)	35 (9.8)
Histology			
SC	176 (17.9)	57 (17.4)	100 (28)
Non-SC	809 (82.1)	270 (82.6)	257 (72)
Grade			
Well-differentiated	1 (0.1)	1 (0.3)	1 (0.2)
Moderately differentiated	144 (14.6)	42 (12.8)	183 (51.2)
Poorly differentiated	792 (80.4)	269 (82.3)	173 (41.4)
Undifferentiated	48 (4.9)	15 (4.6)	0 (0)
Visceral pleural invasion			
No	491 (49.8)	174 (53.2)	NA
Yes	494 (50.2)	153 (46.8)	NA
No. of harvested LNs			
<10	341 (34.6)	113 (34.6)	26 (7.3)
≥10	644 (65.4)	214 (65.4)	331 (92.7)
Skip N2			
Yes	305 (31)	89 (27.2)	120 (33.6)
No	680 (69)	238 (72.8)	237 (66.4)
Involved N2 stations			
Single	492 (49.9)	144 (44)	177 (49.6)
Multiple	493 (50.1)	183 (56)	180 (50.4)
No. of positive LNs			
Median (IQR)	3 (2, 6)	4 (2, 7)	4 (2–8)
LNR			
<0.20	267 (27.1)	83 (25.4)	159 (44.5)
≤0.20 and >0.36	223 (22.6)	69 (21.1)	78 (21.8)
≤0.36 and >0.56	285 (28.9)	93 (28.4)	66 (18.5)
≥0.56	210 (21.3)	82 (25.1)	54 (15.2)
Postoperative chemotherapy			
Without	146 (14.8)	53 (16.2)	42 (11.8)
With	839 (85.2)	274 (83.8)	315 (88.2)
Postoperative chemotherapy			
<4 cycles	247 (25.1)	84 (25.7)	90 (25.2)
≥4 cycles	738 (74.9)	243 (74.3)	267 (74.8)
Postoperative radiotherapy			
Without	689 (69.9)	220 (67.3)	287 (80.4)
With	296 (30.1)	107 (32.7)	70 (19.6)
Adjuvant treatment			
≥4 cycles of POCT with PORT	259 (26.3)	93 (28.4)	58 (16.2)
≥4 cycles of POCT without PORT	479 (48.6)	150 (45.9)	209 (58.5)
<4 cycles of POCT with PORT	37 (3.8)	14 (4.3)	12 (3.4)
<4 cycles of POCT without PORT	210 (21.3)	70 (21.4)	78 (21.9)
Survival information			
No. of recurrence events	668	217	284
Median DFS, months	21.9	22.1	20
95% CI	19.5 to 24.6	18.5 to 24.9	16.8 to 22.3

LNR, lymph node ratio; NA, not available; SC, squamous carcinoma; POCT, postoperative chemotherapy; PORT, postoperative radiotherapy; DFS, disease-free survival; IQR, interquartile range; LN, lymph node.

### Potential Prognostic Factors

The results of the univariable analysis are shown in **Table 2**. The p-values of tumor size, histology, grade, skip N2 (yes or no), involved N2 stations (single or multiple), LNR, and adjuvant treatment pattern were less than 0.1. Larger tumor size, non-squamous cell carcinoma, non-skip N2 disease, multiple N2 stations metastasis, and higher LNR were associated with worse postoperative DFS. Adjuvant treatment pattern was also a factor that had an impact on DFS.

**Table 2:** Univariate analysis, multivariate analysis, and factors for building the adaptive Elastic-Net Cox regression model.

Variable	Univariable analysis	Multivariable analysis			Selected factors for building the model
	p	HR	95% CI	p	β
Age at diagnosis, years	0.4				
<60					
≥60					
Sex	0.3				
Female					
Male					
Smoking history	0.5				
Never smoker or light ex-smoker					
Ex-smoker (non-light)					
Family cancer history	0.5				
Without					
With					
Resection type	0.6				
Lobectomy					
Sleeve resection					
Pneumonectomy					
Tumor size, cm	0.03			0.0038	0.11
≤3		Reference			
>3 and ≤5		1.046	0.8782 to 1.245		
>5		1.436	1.1240 to 1.834		
Tumor location	0.5				
Right upper lobe					
Right middle lobe					
Right lower lobe					
Left upper lobe					
Left lower lobe					
Others					
Histology	0.01			0.0068	0.23
SC		Reference			
Non-SC		1.348	1.0857 to 1.674		
Grade	0.1			0.65	0.01
Well-differentiated					
Moderately differentiated					
Poorly differentiated					
Undifferentiated					
Visceral pleural invasion	0.4				
No					
Yes					
No. of harvested LNs	0.4				
<10					
≥10					
Skip N2	<0.001			<0.001	0.32
Yes		Reference			
No		1.406	1.1514 to 1.716		
Involved N2 stations	<0.001			0.068	0.14
Single					
Multiple					
LNR	<0.001			0.0417	0.09
<0.20		Reference			
≤0.20 and >0.36		1.001	0.7814 to 1.281		
≤0.36 and >0.56		1.292	1.0097 to 1.654		
≥0.56		1.335	1.0090 to 1.765		
Adjuvant treatment	0.01			0.0168	0.10
≥4 cycles of POCT with PORT		Reference			
≥4 cycles of POCT without PORT		1.252	1.0413 to 1.504		
<4 cycles of POCT with PORT		1.743	1.1642 to 2.610		
<4 cycles of POCT without PORT		1.450	1.1489 to 1.831		

LNR, lymph node ratio; SC, squamous carcinoma; POCT, postoperative chemotherapy; PORT, postoperative radiotherapy; HR, hazard ratio; 95% CI, 95% confidence interval; p, p-value; β, coefficient of each factor in the adaptive Elastic-Net Cox regression model; LN, lymph node.

Tumor size, histology, skip N2 (yes or no), LNR, and adjuvant treatment pattern were identified as independent prognostic factors after multivariable Cox regression analyses. Larger primary tumor size and non-squamous cell carcinoma were identified as risk factors of recurrence. With respect to several factors associated with N categories, skip N2 and lower LNR were associated with better prognosis. Patients who finished four or more POCT cycles and received PORT had superior survival. The univariable analysis results, multivariable analyses, and coefficients of each variable entered in the final model are listed in **Table 2**.

### Developing the Prognostic Nomogram for Disease-Free Survival

In order to construct the prognosis model based on the adaptive Elastic-Net Cox regression, variables with p-value <0.1 in the univariate analysis were selected. We compared the predictive performance of the model incorporating all significant variables from the univariable analysis (median AUC: 0.66; range, 0.64 to 0.69) *versus* all independent prognostic factors (median AUC: 0.65; range, 0.64 to 0.68) and found that the model built with significant variables from the univariable analysis performed better.

**Figure 1:**
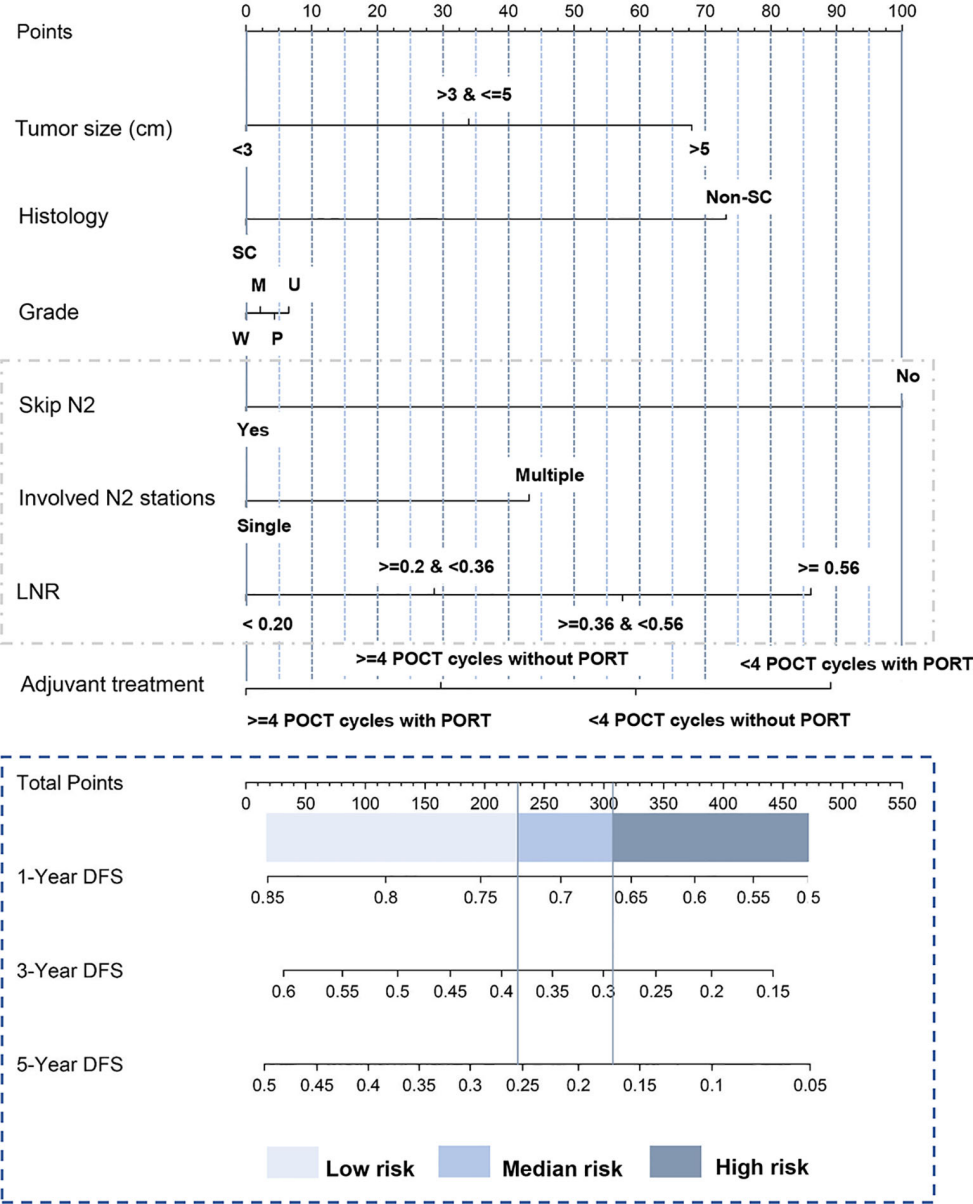
Nomogram predicting postoperative DFS of patients with pathologic N2 non-small cell lung cancer. The tertile of the risk points of patients in the training set was defined as the cutoff value. All patients were divided into three subgroups, named low-risk group (risk point: 0–226), median-risk group (risk point: 226–306), and high-risk group (risk point: 306–466). **Supplementary File 1** provides detailed information for the nomogram usage. SC, squamous carcinoma; Non-SC, non-squamous non-small-cell lung cancer; W, well-differentiated; M, moderately differentiated; P, poorly differentiated; U, undifferentiated; LNR, lymph node ratio. Skip N2 was defined as the tumor “skips” over the N1 (bronchopulmonary or hilar lymph nodes metastasis) stage to N2 (ipsilateral mediastinal lymph nodes metastasis) stage. Lymph node ratio was defined as the number of positive nodes/the number of resected nodes. DFS, disease-free survival.

A nomogram that combined all significant variables from the univariable analysis to estimate the probability of DFS was established in the training set (**Figure 1**). The skip N2 variable demonstrated the largest impact on the prognosis with the highest score among all the factors, followed by adjuvant treatment pattern and LNR (**Figure 1**). The tumor size and histologic type made a moderate contribution to prognosis (**Figure 1**). Each level of these variables was assigned to a point score ranging from 0 to 100 on the point scale. We could estimate 1-year DFS, 3-year DFS, and 5-year DFS individually by adding up points of all variables and drawing a vertical line down to survival scales. The detailed instruction of the nomogram is listed in **Supplementary File 1** (**Figure 1**).

### Calibration and Validation of the Nomogram

**Figure 2:**
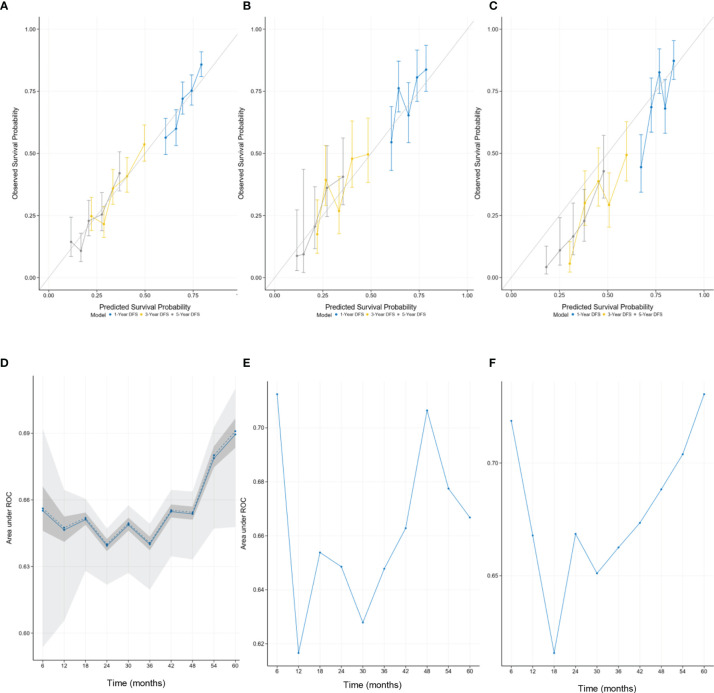
The calibration curves for predicting patient survival at each time point in the **(A)** training set, **(B)** validation set 1, and **(C)** validation set 2. Nomogram-predicted disease-free survival (DFS) is plotted on the x-axis; actual DFS is plotted on the y-axis. A plot along the 45° line would indicate that the predicted probabilities are identical to the actual survival outcomes. The time-dependent AUC showed the median AUC at every 6 months in the **(D)** training set, **(E)** validation set 1, and **(F)** validation set 2. **(D)** The performance of the model in the internal validation. **(E, F)** The performance of the model in the external validation. AUC, area under the receiver operating characteristic curve.

The calibration curves provided good consistency between nomogram prediction and actual observation for 1-year DFS, 3-year DFS, and 5-year DFS in the training set and two validation sets (**Figures 2A–C**). In **Figure 2D**, the time-dependent AUC showed the performance of the model in the internal validation using only the training set data by bootstrap techniques. As shown in **Figure 2D**, the solid line represents the mean of the AUC, and the dashed line represents the median of the AUC. The darker interval shows the 25% and 75% quantiles of AUC, and the lighter interval shows the minimum and maximum of AUC. From the figure, we can see that the bootstrap-based validation result is stable: the median and the mean value at each evaluation time point are close; the 25% and 75% quantiles are also close to the median at each time point. The median AUC was 0.66 (range, 0.64 to 0.69) (**Figure 2D**). **Figures 2E, F** illustrate the performance of the model in the external validation datasets. The median AUC was 0.66 (range, 0.61 to 0.71) in the validation set 1. Similar to validation set 1, the median AUC was 0.67 (range, 0.62 to 0.73) in the validation set 2 (**Figures 2E, F**).

### Discriminative Ability of the Nomogram in Stratifying Risk Groups

**Figure 3:**
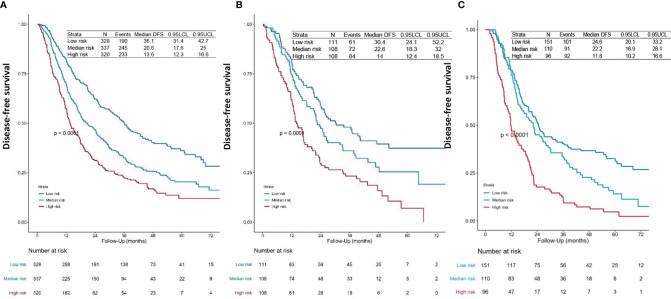
Survival curves. DFS curves for the **(A)** training set, **(B)** validation set 1, and **(C)** validation set 2 of each risk group. The patients-at-risk table is displayed at the bottom of the plots. The risk points for each patient were calculated based on the nomogram (**Figure 1** and **Supplementary File 1**). According to the tertile of the risk points in the training set, all patients were divided into three subgroups, named low-risk group (risk point: 0–226), median-risk group (risk point: 226–306), and high-risk group (risk point: 306–466). DFS, disease-free survival.

We first calculated the risk points for each patient in the training set based on the nomogram. The cutoff values were determined by the tertile of the risk points for patients in the training set. We divided all patients into three subgroups by applying the cutoff value, named low-risk group (risk point: 0–226), median-risk group (risk point: 226–306), and high-risk group (risk point: 306–466). Significant distinctions were demonstrated among different risk groups by the Kaplan–Meier survival curves in the training set (**Figure 3A**), validation set 1 (**Figure 3B**), and validation set 2 (**Figure 3C**).

### Nomogram Predicting Adjuvant Chemoradiotherapy Efficacy

**Figure 4:**
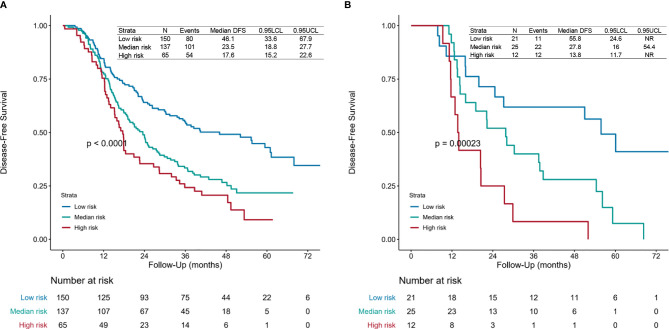
Survival curves. Disease-free survival (DFS) curves for the **(A)** primary cohort (training set and validation set 1) and **(B)** validation cohort (validation set 2) of patients receiving four or more postoperative chemotherapy cycles and postoperative radiotherapy of each risk group. The risk points for each patient were calculated based on the nomogram (**Figure 1** and **Supplementary File 1**). All patients were divided into three subgroups applying the defined cutoff value, named low-risk group (risk point: 0–226), median-risk group (risk point: 226–306), and high-risk group (risk point: 306–466). The patients-at-risk table is displayed at the bottom of the plots. NR, not reached.

Then we calculated the risk points for patients who had finished four or more POCT cycles and PORT using the nomogram (**Figure 1** and **Supplementary File 1**). We categorized those patients into different risk groups by applying the defined cutoff value, low-risk group (risk point: 0–226), median-risk group (risk point: 226–306), and high-risk group (risk point: 306–466). The survival difference between risk groups was statistically significant in the primary cohort (training set and validation set 1) and validation cohort (validation set 2) (**Figures 4A, B**).

## Discussion

Patients with pathologic N2 NSCLC comprise a prognostic heterogeneous group. The optimal treatment for this disease remains a tremendous challenge. Various lymph node parameters reflect N categories from different perspectives and relate to prognosis. In this study, we developed a model that combined detailed lymph node parameters to predict the risk of disease recurrence for patients with resected pN2 NSCLC. By using this model, we could identify patients who remained at a high risk of disease recurrence and make clinical decisions precisely.

The accuracy and reliability of this model were improved by incorporating preoperative, intraoperative, and postoperative variables. Previous studies have shown that tumor size is a common independent prognostic factor for pN2 NSCLC (44, 45). Similar results were observed in our model. Larger primary tumor size was associated with a higher risk of recurrence. Previous studies have identified that histology type is significantly related to prognosis, which is in high concordance with our reports (22, 46). Recurrence was more frequently identified in non-squamous than in squamous cell carcinoma. The details of N categories have been proved to be critical for the survival of patients with locally advanced NSCLC (25, 27, 28). In this study, we also found that skip N2, involved N2 stations, and LNR have an impact on the prognosis and incorporated these variables in our final model. The results of this study show that skip N2 was an independent predictor for better DFS. Single N2 station involvement was significantly associated with better survival outcomes in patients with pN2 disease. LNR is an important prognostic factor for survival outcomes in patients.

As we said, management of patients with locally advanced NSCLC remains controversial (9, 47). Several published studies have proved that adjuvant chemotherapy is associated with better prognosis, while the role of PORT is still not clear (7, 8). In the current study, postoperative data were collected in detail and evaluated strictly, including information about the completion of POCT and PORT. The study found that patients with complete resection who had finished four or more POCT cycles and PORT had superior DFS.

However, we found that a large proportion of these patients remained at high risk of disease recurrence by further dividing the population who completed chemotherapy and radiotherapy.

Our nomogram was constructed and validated based on the data from two separate medical centers with the long-term follow-up of 60 months (**Figure 1**). The model was developed *via* adaptive Elastic-Net Cox regression, which can handle the collinearity problem properly. For a regular multivariate Cox regression analysis, the regression coefficients may have high variance, especially when predictors are correlated to some extent. The adaptive Elastic-Net Cox regression can alleviate this problem by adding a regularization constraint to regression coefficients (32). This method can improve the generalization of the regression model. In this study, variables associated with N categories (skip N2, involved N2 stations, and LNR) were better incorporated in this way.

Validation and calibration were performed to guarantee the robustness of this model. Calibration curves provided optimal agreement between nomogram prediction and actual observation in the training set and two independent validation sets (**Figures 2A–C**). The time-dependent ROC illustrated the discriminatory capability of the nomogram at different time points. The similar discriminative ability between validation set 1 and validation set 2 showed the universality of this nomogram (**Figures 2E, F**).

Limited by the retrospective nature, we failed to incorporate some potential prognostic factors. Due to the influence of culture, medical traditions, and patient willingness, there are still large quantities of patients treated with upfront surgery as first-line therapy in our country, and part of the patients did not receive adjuvant chemotherapy. For statistical reasons, we included the cycles of POCT for analysis instead of excluding patients without POCT. Recent literature has shown that selected patients with stage IIIA NSCLC who received upfront surgery followed by adjuvant therapy may achieve favorable survival outcomes (48). More research is needed for this specific population. Lastly, the model did not perform very well on risk stratification of the validation set 2 (**Figure 3C**). The survival curves of the high-risk group and the median-risk group were not separated (**Figure 3C**). This study was a tentative exploration of predicting individualized prognosis in resected pN2 NSCLC. Our future work is to integrate more potential predictive factors, including biomarkers and radiomics features, to optimize the prognosis model and implement a more precise classification of this population.

## Conclusion

We developed and validated a nomogram that contained multiple lymph node parameters to predict DFS of patients with resected pN2 NSCLC individually. Through this model, we found a subgroup that remained at high risk of disease recurrence after adjuvant chemoradiotherapy. Our finding indicates that traditional chemotherapy and radiotherapy may have reached a bottleneck for a subset of resected pN2 NSCLC patients. Emerging therapy, such as molecularly targeted therapy and immunotherapy, might be a way to improve the survival outcome for selected patients.

## Author's Note

Part of the work was presented at the IASLC 2020 World Conference on Lung Cancer.

## Data Availability Statement

The raw data supporting the conclusions of this article will be made available by the authors, without undue reservation.

## Ethics Statement

The studies involving human participants were reviewed and approved by The Institutional Review Boards of Shanghai Chest Hospital. The patients/participants provided their written informed consent to participate in this study.

## Author Contributions

X−LF, WF, and C-CZ: conceptualization. X−LF, WF, and C-CZ: data curation. C-CZ, R-PH, WF: formal analysis. X−LF: funding acquisition. R-PH and WF: investigation. C-CZ and R-PH: methodology. X−LF: project administration. C-CZ: software. X−LF: supervision. C-CZ and WF: validation. C-CZ: roles/writing—original draft. X−LF, WF, C-CZ, and R-PH: writing—review and editing. All authors contributed to the article and approved the submitted version.

## Funding

This work was supported by the Major Research Plan of the National Natural Science Foundation of China [grant number 92059206], the Shanghai Chest Hospital Project of Collaborative Innovation [grant number YJXT20190101], and the Project of Multi-center Clinical Research, Shanghai Jiao Tong University School of Medicine [grant number DLY201619].

## Conflict of Interest

The authors declare that the research was conducted in the absence of any commercial or financial relationships that could be construed as a potential conflict of interest.

## Publisher’s Note

All claims expressed in this article are solely those of the authors and do not necessarily represent those of their affiliated organizations, or those of the publisher, the editors and the reviewers. Any product that may be evaluated in this article, or claim that may be made by its manufacturer, is not guaranteed or endorsed by the publisher.

## Supplementary Material

Supplementary Figure 1 Consort diagram. NSCLC, Non–small cell lung cancer.
