# Supplementary figures and images for: Lymph Node Parameters Predict Adjuvant Chemoradiotherapy Efficacy and Disease-Free Survival in Pathologic N2 Non-Small Cell Lung Cancer
Source: Front Oncol. 2021 Sep 17;11:736892. doi: 10.3389/fonc.2021.736892 (PMC8484950; doi:10.3389/fonc.2021.736892)

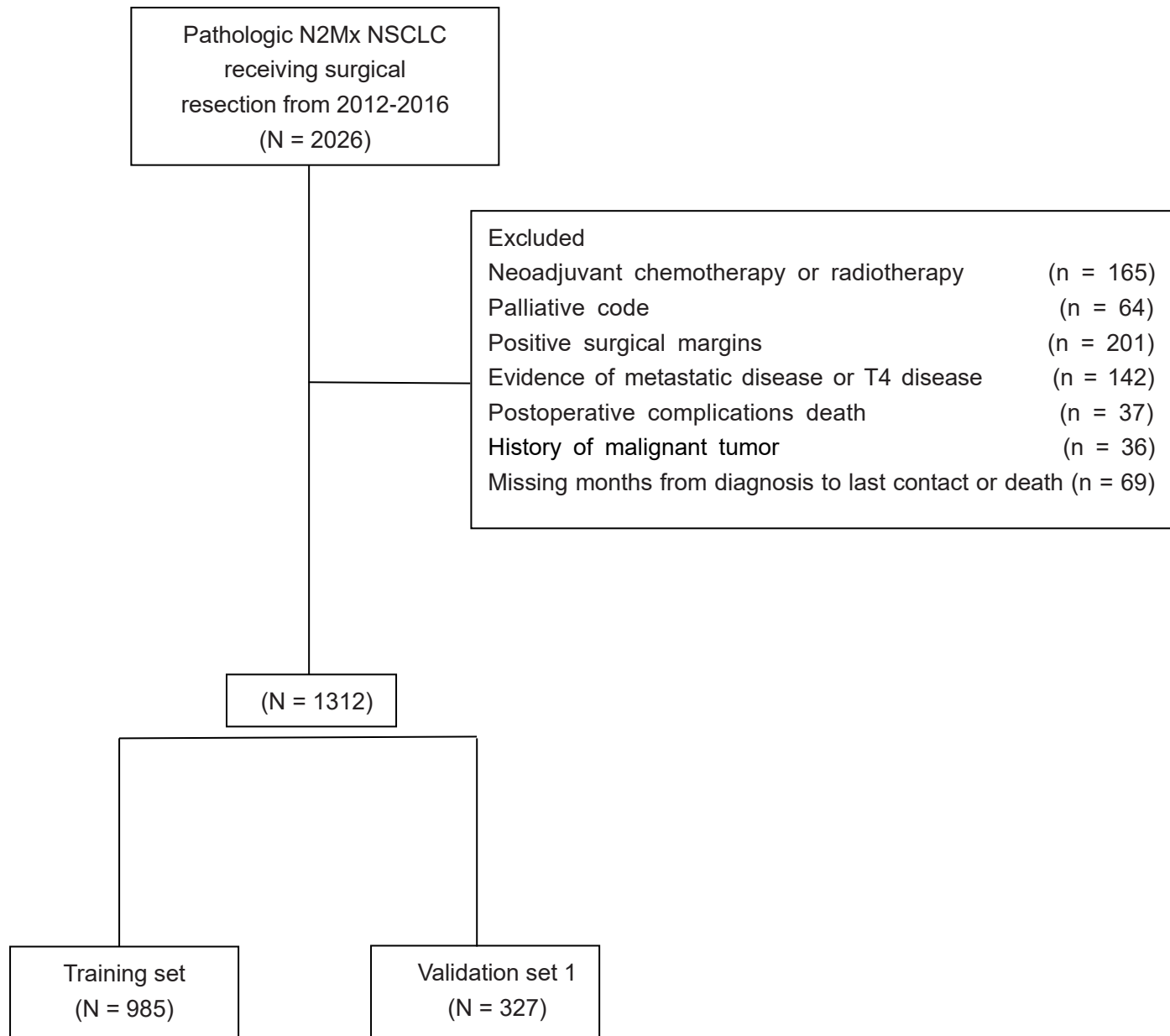

n represents patients excluded; N represents patients left

Supplement: Supplementary Figure 1 — Consort diagram. NSCLC, Non–small cell lung cancer. [file Image_1.pdf]
