# Supplementary material for: Lymph Node Parameters Predict Adjuvant Chemoradiotherapy Efficacy and Disease-Free Survival in Pathologic N2 Non-Small Cell Lung Cancer
Source: Front Oncol. 2021 Sep 17;11:736892. doi: 10.3389/fonc.2021.736892 (PMC8484950; doi:10.3389/fonc.2021.736892)
Supplement: Supplementary file 2 [file Table_1.pdf]

Supplementary File 1

Instructions for using the nomogram

This is an instruction for using the nomogram in Figure 1.

Variables with a P value less than 0.1 in the univariable analyses were used to build the adaptive Elastic-Net Cox regression model. The nomogram is used to visualize the model. According to the contribution degree (regression coefficient) of each factor in the model, the value level of each factor is assigned. Each level of these variables was assigned to a point score ranging from 0 to 100 on the point scale.

Primary tumor size was categorized as less than 3 cm (<=3; score=0), more than 3 cm and less than 5 cm (>3 & <=5; score=34), more than 5 cm (>5; score=68).
Histology was dichotomized as squamous carcinoma (SC; score=0) and non-squamous non-small-cell lung cancer (Non-SC; score=73).
The grade was categorized as Well-differentiated (W; score=0), Moderately differentiated (M; score=2), Poorly differentiated (P; score=4), and Undifferentiated (U; score=6).
Skip N2 was defined as the tumor “skips” over the N1 (bronchopulmonary or hilar lymph nodes metastasis) stage to N2 (ipsilateral mediastinal lymph nodes metastasis) stage and categorized as skip N2 disease (Yes; score=0) and non-skip N2 disease (No; score=100).
The involved N2 station was categorized as single station (score=0) and multiple stations (score=43).
Lymph node ratio (LNR) was defined as the number of positive nodes/the number of resected nodes and categorized as <0.2 (score=0), <=0.20 & >0.36 (score=29), <=0.36 & >0.56 (score=58), and >=0.56 (score=87).
The pattern of adjuvant treatment included >= 4 POCT cycles with PORT (score=0), >= 4 POCT cycles without PORT (score=29), <= 4 POCT cycles without PORT (score=59), <= 4 POCT cycles with PORT (score=89).

Users could estimate 1-year DFS, 3-year DFS, and 5-year DFS individually by adding up points of all variables and drawing a vertical line down to survival scales.

*Low-risk group (risk point: 0-226), median-risk group (risk point: 226-306), and high-risk group (risk point: 306-466) were marked with different colors.
